# Imaging Approaches for the Diagnosis of Dry Eye: A Review

**DOI:** 10.3390/diagnostics16010126

**Published:** 2026-01-01

**Authors:** Angelo Macri, Margherita Tarallo, Michele Iester

**Affiliations:** 1 IRCCS Ospedale Policlinico San Martino, 16132 Genoa, Italy; 2 Department of Neuroscience, Rehabilitation, Ophthalmology, Genetics, Maternal and Child Health (DiNOGMI), University of Genoa, 16132 Genoa, Italy; marghe.tarallo@gmail.com (M.T.); michele.iester@unige.it (M.I.)

**Keywords:** dry eye disease, imaging techniques, diagnosis and management of DED

## Abstract

Dry eye disease (DED) is a multifactorial disorder of the ocular surface, characterised by tear film instability, hyperosmolarity, inflammation, and neurosensory abnormalities. Its clinical heterogeneity and the weak correlation between symptoms and signs complicate both diagnosis and management. Conventional assessments, such as patient-reported symptom questionnaires and basic clinical tests like the Schirmer test, are useful; however, their variability and limited sensitivity highlight the need for more reliable and objective diagnostic tools. This narrative review summarises and analyses current imaging approaches used for the diagnosis of DED. A comprehensive literature search was performed in the PubMed and Google Scholar databases to identify relevant studies published up to October 2025. In recent years, imaging technologies have revolutionised the approach to DED. Modalities such as in vivo confocal microscopy (IVCM), meibography, anterior segment optical coherence tomography (AS-OCT), interferometry, thermography, tear fluorescein clearance, impression cytology, and multifunctional imaging systems allow for non-invasive, high-resolution, and reproducible assessment of ocular surface structures and tear film dynamics. The integration of these techniques into clinical practice supports a more personalised management of DED. Future directions include further technological refinements and the application of artificial intelligence (AI) to imaging analysis, with the potential to enhance diagnostic precision and facilitate earlier intervention. While imaging cannot replace a thorough clinical examination, it has become an essential adjunct that significantly enriches the evaluation and management of patients with DED.

## 1. Introduction

Dry Eye Disease (DED) is a multifaceted condition that significantly affects the quality of life for millions of individuals worldwide [1]. It is a highly prevalent, multifactorial disorder of the ocular surface that results in tear film instability, hyperosmolarity, inflammation, and neurosensory abnormalities [2]. This instability triggers inflammation and damage to the ocular surface, as well as altered nociceptive responses, which collectively cause ocular discomfort and reduce visual function [3].

The eyelids play a crucial role in maintaining the ocular surface system, as they directly influence tear film regeneration, stability, and clearance, while also protecting the ocular surface and indirectly supporting neuroendocrine–immune functions mediated by the tear film. Alterations in eyelid structure or function can disrupt this balance, leading to ocular surface system failure and potentially causing or aggravating DED [4]. Common symptoms of DED include constant itching, redness, dryness, and a burning feeling, often accompanied by a persistent foreign body sensation, light sensitivity, and blurred vision [2].

In epidemiological studies, the prevalence of DED among individuals over 40 years of age ranges from 5% to 50%, with the condition appearing more commonly in older adults and particularly in women. The wide variability in reported prevalence rates is likely attributable to differences in definitions, diagnostic criteria, and study populations across the literature [5].

Despite its frequency, DED remains a complex condition to diagnose and manage, due to the heterogeneity of symptoms and the limited correlation between patient-reported complaints and clinical signs [6]. DED has been shown to negatively affect overall quality of life (QoL), impacting physical, social, and psychological functioning. It can interfere with daily activities such as driving, reading, using a computer, watching television, working, and engaging in social interactions [7].

Accurate diagnosis and effective management are essential to mitigate symptoms and prevent long-term complications [8]. Imaging technologies have revolutionised the field of ophthalmology by providing detailed, non-invasive methods to evaluate the ocular surface and tear film [9]. These advanced imaging modalities offer clinicians valuable insights into the structural and functional aspects of the eye, enabling a more comprehensive understanding of DED pathophysiology. The integration of imaging techniques into clinical practice enhances the diagnostic precision of DED diagnosis, monitors disease progression, and guides therapeutic interventions [10].

Traditional methods of diagnosing DED, such as patient-reported symptom questionnaires and basic clinical tests like the Schirmer test, provide limited insight into the underlying mechanisms and severity of the disease. In contrast, imaging technologies offer detailed, objective, and reproducible evaluations of ocular structures and tear film dynamics [11].

The aim of this review is to provide a comprehensive overview of the current imaging approaches used in the diagnosis of DED, highlighting their principles, diagnostic performance, and clinical applications. By comparing the strengths and limitations of each modality, this work seeks to outline how imaging technologies contribute to a more objective, reproducible, and mechanism-based evaluation of DED, ultimately supporting personalised patient management and improved clinical outcomes.

## 2. Method of Literature Search

This narrative review was conducted to summarise and analyse current imaging approaches used in the diagnosis of DED. A comprehensive literature search was performed in the PubMed and Google Scholar databases to identify relevant studies published up to October 2025. The search strategy combined the following keywords and MeSH terms: “dry eye”, “meibomian gland”, “tear film”, “imaging”, “OCT”, “meibography”, “interferometry”, “topography”, “in vivo confocal microscopy”, “impression cytology”, and “artificial intelligence.” Boolean operators (AND, OR) were applied to optimise the query.

Two independent reviewers screened the titles and abstracts to identify studies relevant to imaging-based diagnostic methods for DED. Eligible articles included original research papers, clinical studies, and reviews focusing on the use of imaging technologies in the evaluation or diagnosis of DED. Studies unrelated to imaging or focused solely on therapeutic interventions were excluded.

The full texts of selected articles were analysed to extract information on imaging modality, study design, population characteristics, diagnostic criteria, and main findings. Data were then categorised according to the imaging technique—such as meibography, anterior segment optical coherence tomography (AS-OCT), interferometry, and in vivo confocal microscopy (IVCM)—and their diagnostic relevance in DED.

Disagreements between reviewers were resolved through discussion and consensus. The final synthesis emphasises advances, diagnostic performance, and clinical applicability of each imaging modality.

## 3. Tear Fluorescein Clearance

Tear turnover, or tear clearance, is described as a global measure of the integrity of the lacrimal functional unit and the efficiency of tear exchange on the ocular surface [12]. The tear turnover rate (TTR) is a time-based parameter reflecting the combined influence of several processes, including tear secretion from the glands, fluid movement through the conjunctiva, tear drainage via the nasolacrimal duct, tear evaporation, and the permeability of the conjunctiva and cornea. TTR has been identified as an indirect marker of ocular surface irritation, the severity of ocular surface disease, meibomian gland dysfunction (MGD), aqueous tear deficiency (ATD), and decreased ocular surface sensitivity [13] Moreover, factors such as eyelid malposition and eyelid laxity can also negatively affect tear turnover by altering the normal dynamics of tear distribution and drainage across the ocular surface [14].

The most widely used techniques for tear turnover assessment are based on following the elution of a tracer molecule added to the tear film using electromagnetic detection methods. Fluorophotometry and lacrimal gamma scintigraphy are the most common techniques. Fluorophotometry involves instilling a small amount of sodium fluorescein into the tear film and measuring the decrease in fluorescence over time to calculate the TTR. The technique requires specialized equipment and trained operators [13].

**Figure 1:**
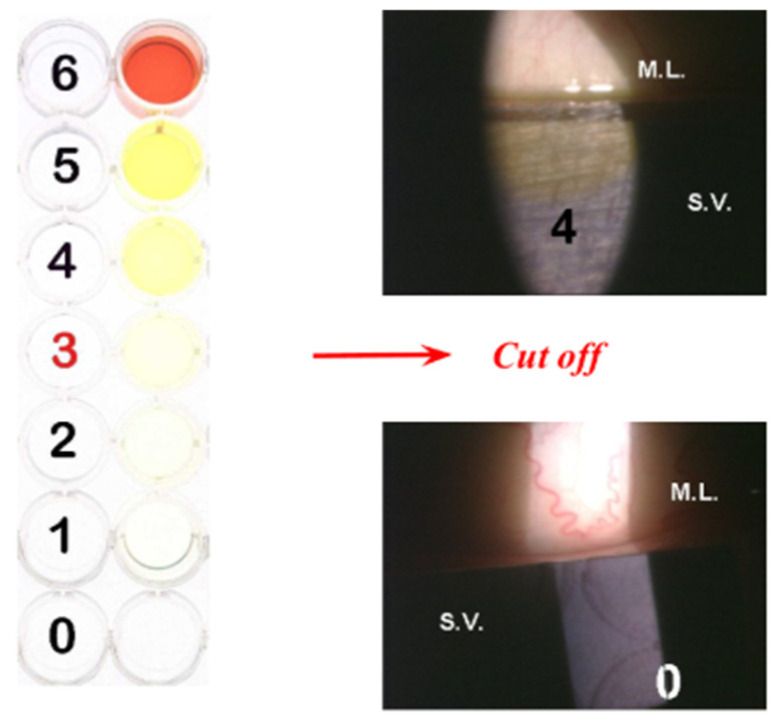
Standardized scale for the evaluation of fluorescein clearance; higher values indicate a more pronounced delay in tear turnover [15].

Although fluorophotometry has been valuable in research settings for establishing the relevance of tear fluorescein clearance as a risk factor for ocular irritation and ocular surface disease, it is not practical for routine clinical use. A standardised visual scale for evaluating tear fluorescein clearance has been validated against fluorophotometry in both healthy individuals and patients with MGD, ATD, or both. The visual scale shows comparable sensitivity and specificity for detecting delayed tear clearance and correlates with ocular irritation symptoms as well as eyelid and corneal epithelial disease (**Figure 1**).

This simple and cost-effective method is suitable for routine clinical use, allowing broader implementation of tear clearance assessment and helping to identify patients who may benefit from pharmacologic treatment for ocular surface inflammation associated with delayed tear clearance [15].

In addition to traditional methods like fluorophotometry, tear turnover can also be evaluated using AS-OCT. This technique allows precise measurement of tear meniscus morphology, including tear meniscus height (TMH), depth (TMD), and cross-sectional area (TMA), with high repeatability and the ability to track post-instillation changes [13]. This is described in the following paragraph.

## 4. AS-OCT

**Figure 2:**
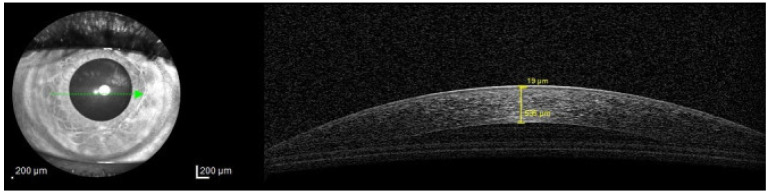
Tear film thickness measurement technique. From surface to depth, two values can be seen in every image: the first, superficial one, is of the tear film thickness, while the second one is of the corneal thickness [21].

AS-OCT is a non-invasive imaging technique increasingly used in the evaluation of DED that provides high-resolution cross-sectional images of the anterior segment [11]. While it is often compared to ultrasound, OCT offers higher resolution and does not require direct contact with the eye [16] (**Figure 2**).

In routine clinical practice, AS-OCT is widely used in routine clinical practice to measure and visualize several tear film parameters [11]:

**Tear Meniscus Height (TMH)**: THM is the thin strip of tear fluid found at the lower eyelid margin. A reduced TMH is often indicative of tear deficiency [17]

**Pre-corneal Tear Film Thickness (TFT)**: TFT is associated with ocular surface health, though its measurement repeatability is still debated. AS-OCT provides an indirect measurement by comparing corneal thickness before and after contact lens application. Healthy subjects have an average TFT of approximately 5 µm [18]

**Tear Meniscus Curvature (TMR):** The radius of meniscus curvature correlates with tear volume and shows good diagnostic accuracy for DED [19].

**Tear Meniscus Volume (TMV)**: TMV is a good indicator of overall tear volume and secretion rate [20].

AS-OCT is also employed to assess corneal parameters, such as the epithelial thickness profile, and to obtain high-resolution 3D images of the meibomian glands. In MGD patients, it has shown a reduction in gland length and width [12].

## 5. In Vivo Confocal Microscopy

IVCM is an imaging technique that has significantly enhanced our understanding and management of DED. This technology allows for high-resolution, in vivo visualisation of the corneal and conjunctival microstructures at a cellular level, providing invaluable insights into the pathophysiology of DED [22]. The principle of confocal microscopy lies in the conjugate alignment of light rays focused on the tissue with those reflected and transmitted to the observer, hence the term “confocal” [10].

IVCM is used to assess microstructural changes in the cornea, particularly in epithelial cells, keratocytes, and corneal nerve fibres [23].

Studies have shown significant alterations in the corneal epithelium of DED patients, including the presence of hyper-reflective cells. These changes are thought to result from increased desquamation of superficial cells and inflammation-related alterations caused by tear film hyperosmolarity [24]. A marked reduction in epithelial cell density has also been observed in DED patients compared with healthy controls.

Additionally, a higher density of basal epithelial cells has been observed, reflecting accelerated cell turnover as a compensatory response to the loss of superficial cells. Both superficial and basal epithelial cell densities have been shown to normalise following anti-inflammatory treatment [5,6].

In DED, there is a notable increase in the density and metabolic activity of anterior stromal keratocytes probably as a response to chronic inflammation. Enhanced keratocyte activity can result in abnormal stromal hyper-reflectivity, where keratocytes appear as bright, hyper-reflective cells. This increased brightness is attributed to the elevated metabolic activity of keratocytes under inflammatory stress [24].

**Figure 3:**
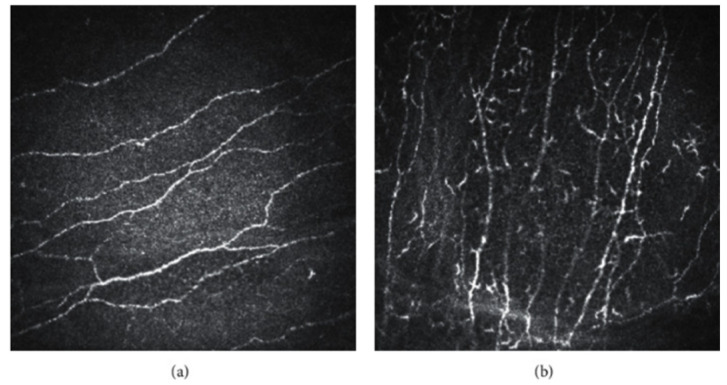
In vivo confocal microscopy. (**a**) Normal sub-basal nerve plexus. (**b**) Increased dendritic cells in dry eye disease [27].

Furthermore, IVCM plays a crucial role in evaluating alterations in corneal nerve density and structure. Abnormalities in the sub-basal nerve may include bead-like formations, tortuosity, irregular branching patterns, neuromas, atypical plexus loops, and inter-neuronal connections. In the early stages of DED, nerves exhibit increased activity and reflectivity in response to inflammation. Tortuosity may also be observed, suggesting longer nerve length. As the disease progresses and inflammation damages the nerves, a decrease in nerve density is expected [25] (**Figure 3**).

IVCM is also valuable for monitoring therapeutic effects, such as the decrease of dendritic cells and the increase of epithelial cell density following treatment [24].

The presence and density of dendritic cells in the cornea can be evaluated using IVCM. An increase in dendritic cell density is typically associated with ocular surface inflammation in DED [26]. Dendritic cells are involved in immune modulation and antigen presentation, influencing pain pathways by interacting with T-helper cells. Studies suggest that corneal dendritic cells might contribute to the development of DED, keratoconjunctivitis sicca, and rejection of corneal transplants. Inflammatory conditions are characterised by a higher number of corneal dendritic cells, particularly in patients with severe symptoms [24].

Recently, IVCM has been frequently employed to investigate MGD, an important factor in the aetiology of DED. This common ocular surface disorder, typically obstructive in nature, results in reduced lipid secretion, increased tear evaporation, and decreased tear stability, leading to ocular surface damage. IVCM can be used to measure morphological changes in the meibomian glands, including the diameters and densities of the acinar units, the diameter of the MG orifices, and the density of periglandular inflammatory cells. In patients with MGD studies observe a decrease in acinar cell density, an increase in acinar unit diameters, and a reflectance increase in meibomian secretions [28].

## 6. Meibography

Meibography is an imaging technique used to assess the structure and function of the MG in vivo. MG play a crucial role in the pathophysiology of evaporative DED [29]. Studies have shown that the reflectivity and contrast of MG can indicate gland function, as reflectivity decreases during isotretinoin treatment and increases after discontinuation [30]. Two main meibography techniques are available: contact and non-contact meibography.

**Contact meibography**: developed in the late 1970s, the traditional method involves the direct application of a light probe to the eyelid to evert and transilluminate the tarsal plate, followed by image capture using a specialised camera [31].

This technique presents several challenges: It requires operator expertise, anatomical variations of the eyelids can cause probe manipulation, and patients often experience discomfort due to heat, pressure, or brightness [7].

**Non-contact meibography**: Introduced in 2008, non-contact meibography uses a slit-lamp biomicroscope with an IR filter and an IR charge-coupled device video camera to image a digitally everted eyelid. By removing the need for a light probe, non-contact meibography avoids the issues of discomfort and technical difficulty associated with contact methods. It is therefore faster, more patient-friendly, and easier to perform. Another advantage of non-contact meibography is its ability to view a larger surface area of the everted eyelids, reducing both the number of images required and the time needed to construct a panoramic view [9,11].

Optical coherence tomographic meibography (OCTM) is the latest advancement in meibography. It was first described by Bizheva et al. in 2010 [32]. OCTM is a non-invasive technique that can capture two- and three-dimensional tomograms of the MG in vivo. Unlike conventional meibography, OCTM uniquely enables the volumetric quantification of MG morphology, a capability that was previously only possible through ex vivo histologic studies [31].

The area of MG dropout was positively correlated with the meibum quality grading [33]. Additionally, both MGD severity and MG dropout were correlated with the tear break-up time (TBUT), dry eye symptoms score, and corneal staining. Ductal length and acinar area, as measured by meibography, also correlate with tear film characteristics, corneal staining, and meibum quality [27].

In clinical practice, grading systems describing MG structural features are used to document the presence, progression, and treatment response to MGD [16].

## 7. Meibographic Image Analysis

In this imaging method, healthy meibum appears bright due to its natural autofluorescence. Conversely, dark areas within the MG may suggest the loss of acinar tissue or changes in meibum consistency, which are indicative of MG dropout [27].

Abnormal MG show features consistent with histopathological studies, in which ducts appear dilated and glands become enlarged, feel tortuous, and eventually drop out [31].

Among the various grading scales proposed for assessing MG dropout, the Gestalt grading scale and meiboscale have been endorsed by the MGD Workshop.

In the Gestalt grading scale, each eyelid is evaluated on a scale from 1 to 4 based on the proportion of partial glands present:

Grade 1: No partial glands.

Grade 2: Less than 25% partial glands.

Grade 3: 25% to 75% partial glands.

Grade 4: Greater than 75% partial glands.

On the other hand, the meiboscale grades each eyelid according to the extent of MG dropout:

Grade 0: No loss of MG.

Grade 1: Area loss ≤ 25%.

Grade 2: Area loss ≤ 50%.

Grade 3: Area loss ≤ 75%.

Grade 4: Area loss ≤ 100%.

These grading scales provide standardized methods to assess the severity of MG dropout, aiding clinicians in diagnosing and monitoring MGD [27].

**Figure 4:**
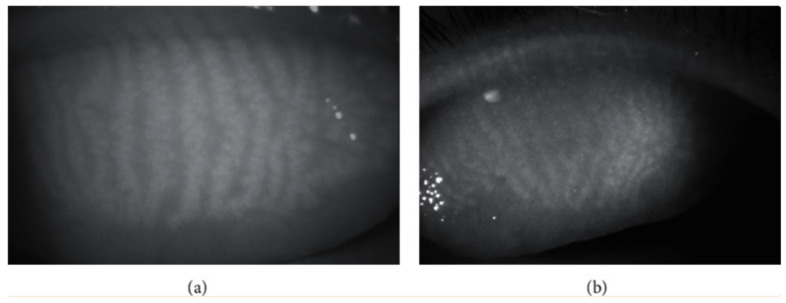
(**a**) Infrared meibography comparison of a healthy subject and a (**b**) patient with dry eye syndrome.

Infrared meibography displays relatively normal MG in the upper eyelid, as seen in **Figure 4a** where the brighter regions correspond to glandular tissue and the darker regions represent interglandular areas. Conversely meibography can illustrate mild atrophy of the MG near the proximal margin of the tarsal plate with the affected area appearing greyish and lacking the characteristic whitish lines that typically indicate healthy glands **Figure 4b** [27].

## 8. Artificial Intelligence (AI) in MG Evaluation

Conventional approaches to analysing MG images and making clinical judgments are limited by their subjective nature and time requirement, making them less practical for routine clinical use. Computer-assisted MG evaluation methods employing AI, including computer vision and machine learning, have been developed to provide more accurate, objective, and efficient assessments.

Significant progress has been made in improving analytical accuracy and enabling the quantification of parameters such as gland count, area, and shape. Computer vision techniques allow for automatic MG detection and segmentation, while supervised learning predicts key parameters, and unsupervised learning identifies hidden structural patterns. These AI-based tools offer clinicians comprehensive visual insights into patients’ ocular surface health [34].

## 9. Non-Invasive Breakup Time (NIBUT)

The NIBUT is a crucial diagnostic tool in assessing the stability and integrity of the tear film on the ocular surface [35].

Unlike traditional methods that require fluorescein dye, NIBUT provides a less invasive means of evaluating tear film stability by using instruments such as corneal topographers or keratometers. These devices use reflective patterns or infrared imaging to detect the first signs of tear film disruption.

The values of fluorescein BUT can vary based on factors such as the amount, concentration, pH, drop size, presence of preservatives, and the type of fluorescein used. Additionally, BUT measurements can be influenced by ocular surface irregularity, lighting techniques, and reflex tearing induction [27].

Many of these techniques rely on observing the specular reflection of an illuminated grid pattern on the tear film, often resulting in longer measured tear breakup times compared to fluorescein methods. NIBUT can also be assessed by analysing the placido disk images’ reflections from the anterior ocular surface, a feature available in many modern corneal topography systems.

Automated assessment of tear film stability is also possible with specific software such as the Keratograph (Oculus, Wetzlar, Germany), which detects and maps the locations of tear breakup over time. A standardised methodology is important, with instructions to blink naturally three times and then to stop blinking until instructed.

The NIBUT sensitivity and specificity differ depending on the specific technique employed, with reported sensitivity ranging from 82% to 84% and specificity from 76% to 94%. Generally the cutoff value of 10 s or less has been identified as indicative of DED [36].

AI-based systems represent a promising alternative, enabling automated TBUT measurement through video and image analysis. Deep learning algorithms can detect tear film break-up points, analyse blink dynamics, and predict instability by integrating multimodal clinical data. These technologies help reduce observer bias and enhance diagnostic accuracy [37].

## 10. Interferometry

Interferometry is a valuable instrument in the diagnosis and management of DED [38]. In this context, the technique is primarily used to analyse the tear film [39].

The outermost layer of the tear film, composed of lipids, helps to stabilise the distribution of the tear film and minimises its evaporation. Additionally, this lipid layer prevents the overflow of aqueous tears onto the eyelids [40].

Interferometry can be employed to examine the transparent lipid layer on the surface of the tear film. A thinner or disrupted lipid layer can lead to increased evaporation and dry eye symptoms [41].

These techniques is also used to evaluate how lipid layer spread after a blink [42]. It has been observed that the time it takes for the interference pattern to stabilize after a blink, known as spreading time, longer in eyes with an aqueous deficiency compared to those with adequate aqueous content [43]. In healthy eyes, lipid layer thickness has been estimated and reported to be approximately 70 nm [11].

An interferometer operates by merging two distinct light beams using the principle of superposition. In ophthalmic interferometry, these light beams are reflected off the front and back surfaces of a transparent medium, like the tear film. They are then combined again at the detector of the device, where the variation in the beams’ paths provides the diagnostic measurement [39].

In summary, interferometry is a non-invasive and precise method for assessing the tear film in dry eye patients [44].

## 11. Thermography

Thermography is a non-invasive imaging technique that measures the temperature of the skin or surface tissues [45]. It is particularly useful for studying ocular surface temperature (OST) in relation to DED [46].

Thermography allows for an indirect assessment of the evaporation rate by noninvasively measuring the OST with a thermographic camera that functions in the infrared range [47,48].

The ability to monitor OST in real time provides advantages for studying post blink changes in temperature. Since the cornea does not transmit infrared radiation beyond 2.3 µm, it is considered an efficient radiator, making it suitable for comparison with a perfect black body.

The tear film, with its high water content, plays a crucial role in absorbing and radiating infrared radiation, meaning that the temperature measured is primarily tear film temperature. Deeper ocular structures contribute minimally to the detected radiation due to their absorption characteristics. Factors like blinking, tear film thinning, and environmental conditions can influence OST measurements.

In dry eyes, the average OST tends to be higher, with a sharper thermal gradient from the centre of the cornea to the limbus, and the surface cools more quickly after each blink [49]. This is likely due to decreased tear film stability and to a higher evaporation rate [48,50].

Immediately after eye opening, the ocular surface temperature in dry eyes is comparable to that of normal eyes. However, after 10 s, the temperature drop in patients with DED becomes significantly more pronounced than in healthy individuals. Additionally, this reduction in corneal temperature has been shown to correlate strongly with the TBUT [51].

This real-time feedback is one of thermography’s key advantages. As a non-invasive and rapid imaging technique, it allows clinicians to immediately assess how a patient is responding to treatments such as artificial tears [46].

## 12. Impression Cytology (IC)

IC is a minimally invasive technique used to collect superficial epithelial cells from the conjunctiva and cornea. It provides cytological proof for the diagnosis of various ocular surface disorders [52].

Conjunctival impression cytology (CIC) represents an important diagnostic tool for assessing cellular morphology and goblet cell density (GCD). Patients with DED exhibit significantly lower GCD compared with healthy controls. Furthermore, ocular surface disorders are frequently associated with morphological alterations of conjunctival epithelial cells, in addition to changes in conjunctival goblet cells (CGCs) [53].

The procedure involves placing a filter paper, such as an acetate filter paper, on the bulbar or palpebral conjunctiva and applying slight pressure to induce the adhesion of superficial 1–3 layers while the patient is under local anaesthesia. The removed sample is then fixed with ethanol or formaldehyde and observed using different methods in combination with a specific experimental purpose [53].

The normal ocular surface is composed of a stratified nonkeratinizing epithelium and, in case of conjunctiva, different amounts of goblet cells, which produce the mucous part of the tear film. Pathological changes, such as squamous metaplasia of the epithelial cells, keratinization, and reduction in GCD, occur in numerous disorders including dry eye, Sjögren’s syndrome, blepharoconjunctivitis, inflammatory keratoconjunctivitis, and vitamin A deficiency [54].

Nelson et al. introduced a grading system for conjunctival squamous metaplasia based on the evaluation of epithelial and conjunctival CGCs changes in CIC. They assessed both the morphology and nucleus–cytoplasm ratio of epithelial cells, as well as the cell morphology and density of CGCs [55].

Although CIC offers valuable insights into ocular surface pathology, its clinical utility in diagnosing DED is limited by considerable overlap in GCD between healthy and diseased eyes and variability in sampling procedures, reducing its diagnostic reliability. Therefore, CIC is more useful as a research tool than as a routine clinical test [53].

## 13. Multifunctional Imaging Device and AI

Multifunctional imaging devices are sophisticated tools that integrate various diagnostic modalities to provide a comprehensive assessment of the ocular surface, tear film, and meibomian glands. These devices are essential in diagnosing, monitoring, and managing DED, as they allow clinicians to evaluate the condition from multiple perspectives. By providing detailed insights, they guide more effective and personalised treatment strategies [37].

The integration of AI in ocular surface imaging represents a fundamental shift from clinician-dependent subjective grading to objective, reproducible quantification. Furthermore, AI facilitates a “mechanism-based” approach to the disease. By processing vast amounts of data at high speeds, AI-assisted systems move beyond mere diagnosis, enabling the identification of subtle structural biomarkers—such as early changes in meibomian gland architecture or micro-fluctuations in tear film stability—which are essential for the development of personalised treatment protocols and the longitudinal monitoring of therapeutic efficacy [34].

## 14. Brief Discussion

Imaging modalities offer reproducible, objective, and highly detailed assessments of both structural and functional alterations associated with DED. As illustrated in **Table 1**, while these technologies allow clinicians to characterise DED with greater precision and monitor disease progression more accurately, certain challenges remain. The high cost of equipment, the requirement for specialise training, and the time needed for sophisticated image analysis can hinder the widespread adoption of these tools in every clinical setting. Furthermore, imaging findings should not be interpreted in isolation.

Despite the high resolution of IVCM or the precision of AS-OCT, these results must always be integrated with a thorough patient history and a traditional clinical examination. The lack of a “gold standard” in DED diagnosis means that a multimodal approach—combining symptoms, clinical signs, and imaging data—remains the most effective strategy for accurate diagnosis.

The future of DED imaging is closely linked to the integration of AI. Automated segmentation and grading systems, particularly in meibography and NIBUT analysis, are expected to reduce observer bias and significantly decrease the time required for data interpretation. Moreover, the development of multifunctional devices that can perform several non-invasive tests in a single session will likely improve patient workflow and diagnostic throughput.

## 15. Conclusions

In conclusion, ocular imaging has become an indispensable complement to conventional clinical assessment in DED. As technology becomes more accessible and automated, imaging will continue to play a pivotal role in improving the quality of life for patients suffering from this complex and multifactorial disease.

## Figures and Tables

**Table 1:** Comparison of imaging techniques for the diagnosis of DED.

Imaging Technique	Main Diagnostic Parameters	Advantages	Limitations	Clinical Usefulness
**TFC**	Tear clearance time, tear film stability.	Simple, inexpensive, validated visual scale available.	Operator-dependent, limited reproducibility.	Useful for screening for delayed tear clearance and ocular surface inflammation.
**AS-OCT**	TMH, TMV, TFT, Corneal Epithelial Profile.	High-resolution, non-contact, reproducible.	Costly equipment, interpretation expertise needed, limited TFT repeatability.	Quantitative evaluation of tear film.
**IVCM**	Epithelial integrity, keratocyte activity, nerve density, dendritic cells.	Cellular-level visualization, detects inflammation and nerve changes, monitors therapy.	Small field of view, time-consuming, operator expertise required.	Detailed evaluation of ocular surface pathology and inflammation in DED.
**Interferometry**	Lipid layer thickness, spreading time, TBUT.	Non-invasive, analyses tear film dynamics and lipid quality.	Sensitive to environment, specialized devices needed.	Quantifies lipid deficiency and evaporation-related dry eye.
**Thermography**	OST gradients, post-blink temperature decay.	Rapid, non-contact, real-time monitoring.	Influenced by environment and blinking, limited availability.	Detects evaporative instability and evaluates treatment response.
**IC**	GCD, epithelial morphology.	Minimally invasive, provides cytological confirmation of ocular surface alterations, useful in research for evaluating cellular changes and goblet cell loss.	Overlap of GCD between healthy and DED eyes, variability in sampling, limited diagnostic reliability, requires laboratory processing.	Valuable for studying ocular surface pathology and monitoring therapeutic effects, primarily a research tool rather than a routine diagnostic test.
**Multifunctional Imaging Devices**	Integrated tear film and gland parameters.	Comprehensive evaluation, efficient workflow, AI integration possible.	High cost, complexity, limited accessibility.	Personalized management of DED.

## Data Availability

No new data were created or analyzed in this study. Data sharing is not applicable to this article.

## Abbreviations

TFC	tear fluorescein clearance
AS-OCT	anterior segment optical coherence tomography
TMH	tear meniscus height
TMV	tear meniscus volume
TFT	pre-corneal tear film thickness
IVCM	in vivo confocal microscopy
DED	dry eye disease
TBUT	tear film break up time
OST	ocular surface temperature
IC	impression cytology
GCD	goblet cell density
AI	artificial intelligence
